# Implementation of Competency-Based Pharmacy Education (CBPE)

**DOI:** 10.3390/pharmacy5010010

**Published:** 2017-02-21

**Authors:** Andries Koster, Tom Schalekamp, Irma Meijerman

**Affiliations:** 1 Department of Pharmaceutical Sciences, Utrecht University, The Netherlands and European Association of Faculties of Pharmacy (EAFP), Utrecht 3508 TB, The Netherlands; 2 Department of Pharmaceutical Sciences, Utrecht University, Utrecht 3508 TB, The Netherlands; T.Schalekamp@uu.nl; 3 Department of Pharmaceutical Sciences, The Netherlands and Centre for Teaching and Learning, Utrecht University, Utrecht 3508 TB, The Netherlands; I.Meijerman@uu.nl

**Keywords:** assessment, competence, competency-based education, constructive alignment, curriculum, development, entrustable professional activity, learning outcomes

## Abstract

Implementation of competency-based pharmacy education (CBPE) is a time-consuming, complicated process, which requires agreement on the tasks of a pharmacist, commitment, institutional stability, and a goal-directed developmental perspective of all stakeholders involved. In this article the main steps in the development of a fully-developed competency-based pharmacy curriculum (bachelor, master) are described and tips are given for a successful implementation. After the choice for entering into CBPE is made and a competency framework is adopted (step 1), intended learning outcomes are defined (step 2), followed by analyzing the required developmental trajectory (step 3) and the selection of appropriate assessment methods (step 4). Designing the teaching-learning environment involves the selection of learning activities, student experiences, and instructional methods (step 5). Finally, an iterative process of evaluation and adjustment of individual courses, and the curriculum as a whole, is entered (step 6). Successful implementation of CBPE requires a system of effective quality management and continuous professional development as a teacher. In this article suggestions for the organization of CBPE and references to more detailed literature are given, hoping to facilitate the implementation of CBPE.

## 1. Introduction

If you want to grow a worthwhile plant: a rose, a fruit tree, a vine of paan, then you need effort.

  *You must water, apply manure, weed it, prune it.*

It is not simple.

  *So it is with the world.*

Vikram Seth: A suitable boy

National and international tendencies indicate that competency-based educational models are becoming dominant for the education of heath care professionals, such as nursing [1], dentistry [2], medicine [3], and pharmacy [4,5]. The main driver for adopting competency-based educational designs is the need to prepare pharmacists for their societal role, ultimately leading to improvement of health care and patient safety [6,7,8,9]. However, implementation of a competency-base pharmacy curriculum is a formidable task, in particular if an existing curriculum is organized in a disciplinary, content-driven, teacher-centered way, in which students are expected to mainly attend lectures and to perform in well-structured practical exercises reproducing compounding and analytical tasks as described in national formularies of pharmacopoeias. Even though this description of a ‘traditional’ curriculum can be considered stereotypical, most readers will recognize elements of this description in their local pharmacy curricula. An additional problem in developing a pharmacy curriculum is the recent change in the tasks of the pharmacist, which, during the last decades, has shown a shift from product-orientation to more patient-orientation (FIP 2012, [4]). Transforming a ‘traditional’ educational practice into competency-based pharmacy education (CBPE), which pays attention to both the science-based and patient-oriented aspects of pharmacy in a balanced way, will involve re-thinking of the roles of teachers, the roles of students, and re-designing of assessment tasks and many educational activities [10,11]. Moreover, a pharmacy department or faculty is usually organized along disciplines ranging from medicinal chemistry, via biopharmacy to pharmacotherapeutics and social pharmacy. It is, therefore, necessary to create a curriculum management structure and a human resources allocation model, which may interfere or conflict with existing hierarchies and research interests. 

This paper describes the essential steps in designing a competency-based pharmacy curriculum and gives tips for a successful organization, development, and implementation of such curricula. Suggestions will be based on literature references whenever possible, but will also be ‘colored’ by the authors’ experiences with implementing new curricula in the field of pharmacy and pharmaceutical sciences [12,13]. Readers should be aware that the possibility to make radical changes in existing curricula depend heavily on the local situation, in particular with respect to the experienced need for change, the preparedness to embark on a complicated journey, and the willingness of the formal departmental and/or university structure to support and facilitate the change process. The experiences of the authors are ‘colored’ by the way a curriculum renewal was handled in a positive and stimulating way by the departmental leadership (cf. [14]). It is, therefore, uncertain whether all aspects, which refer to the authors’ own experiences, can be easily implemented in other environments. Nevertheless, we hope that this article can be a guide in starting an interesting journey towards competency-based pharmacy education (CBPE).

## 2. Competency-Based Pharmacy Education

The attention for competency-based pharmacy education is relatively recent, compared to other health care professional programs. The American Association of Colleges of Pharmacy has pioneered the development of educational outcome-based guidelines since the early 1990s (AACP 2013, [10]) and a global competency framework was published by the International Pharmaceutical Federation more recently (FIP 2012, [4]). Descriptions of the entry-into-practice requirements for professional pharmacists are available for Canada (AFPC 2010), the United Kingdom (GPhC 2011), Australia (NCSF 2010 [15]), and Europe (EPCF 2016, [9]). These descriptions can be based on different models and may have more or less official legal status, but they all intend to function as guiding principles for the evaluation and ‘re-engineering’ of existing curricula and the design and development of new curricula. The requirements for entry-level pharmacists are usually defined in terms of learning outcomes or competencies and are ordered on the basis of Miller’s pyramid of clinical competence [16]. The diversity of frameworks (see Appendix A) illustrates that no ‘golden standard’ for a competency framework exists. As long as the framework is internally consistent and captures all aspects of the required professional competence, it can be used as a tool for the analysis, development, and structuring of a curriculum. Existing frameworks for pharmacy education appear to be similar across jurisdictions [17] and health care competency frameworks in general appear to address the same aspects of professional competence. The use of competency standards for undergraduate pharmacy education was recently reviewed by Nash et al. [5].

Apart from competency frameworks covering the complete initial Pharmacy higher education program, competency profiles have been developed for separate curriculum domains, e.g., advanced pharmacy practice [18], professional development skills [19], or related specialization areas, such as clinical pharmacology [20] or pharmaceutical medicine [21].

## 3. Terminology and Definitions

The implementation of CBPE is often complicated by concepts and terminology, which is experienced as ill-defined or confusing [22,23]. Medical competence is defined as “The array of abilities across multiple domains or aspects of physician performance in a certain context. Statements about competence require descriptive qualifiers to define the relevant abilities, context, and stage of training. Competence is multi-dimensional and dynamic. It changes with time, experience, and setting” (cited from [3], (p. 641)). We suggest that the same definition can be used for other health care professionals, including pharmacists, by changing the use of ‘physician’ into any other relevant professional job description.

The definition of competence makes clear that competence of a student or pharmacist can only be observed or assessed in the context of specific well-defined circumstances. Being competent in one professional situation does not necessarily imply competence in another situation (competence is *contextual*), and students need time to become competent in different aspects of their intended profession (competence is *developmental*). Moreover, it is highly unlikely that all students can acquire competence at the same rate and with the same amount of training provided; large inter-individual differences are usually encountered. Finally, competence is only demonstrated when all relevant knowledge, skills, and behavior is used in an integrated way which is relevant in a particular professional situation (competence is *multidimensional*). The contextual, developmental, and multidimensional nature of the competence to be achieved by a curriculum has important consequences for the organization of CBPE, in particular with respect to assessment and progression of students through the curriculum [3,24]. 

An approach to deal with the complex nature of CBPE is to use *entrustable professional activities* (EPAs) for the operationalization of educational outcomes at the transition of undergraduate education to professional working life. EPAs are carefully described aspects of professional acting, respecting the contextual and developmental aspects of competence, which are used to structure learning, training, and assessment of starting professionals enrolled in medical specialization programs [23]. By proposing the use of EPAs as a way of structuring medical education at an earlier stage, the undergraduate curriculum, medical educators intend to ease the abrupt transition from undergraduate to graduate education [24]. In this conception, undergraduate education, entry into professional life, further specialization, and postgraduate training become a flexible educational continuum where training and assessment is structured by using EPAs as building blocks of competence. In the context of pharmacy education, EPAs are used for structuring the advanced pharmacy practice experience of the University of Minnesota College of Pharmacy, USA [25] and the postgraduate ‘advanced community pharmacy’ specialization in the Netherlands [26].

Competence can be conceptualized as consisting of various ingredients, or building blocks, which together enables the student to function in a competent way. These building blocks of competence are designated as competencies (singular: *competency*). Competencies are preferably specified as observable abilities of a pharmacist, integrating multiple components such as knowledge, skills, values, and attitudes, and expressed as actual behavior. Since competencies are observable, they can be measured and assessed to ensure that students have acquired them [3]. Moreover, progression of students through the curriculum can be guided and monitored by defining intermediate stages in the acquirement of competencies (see below). These intermediate stages can be used as anchor points to structure the curriculum and/or as critical points for assessing whether students are progressing according to expectations. Competencies are acquired by the students while they progress through the curriculum and must be considered a personal qualities or abilities of the student [23]. 

In order to guide the development of assessment formats and teaching-learning activities (see below) competencies usually need to be further broken down in their constituent elements. Most competencies, as defined in existing competency frameworks, each consist of a unique mixture of knowledge in particular disciplines, cognitive skills, non-cognitive skills, and attitudinal aspects, which need to be used or applied in an integrated way. In undergraduate education different fields of knowledge (disciplinary or otherwise) and a variety of skills (technical, cognitive, non-cognitive, etc.) can be taught or trained in several ways, but assessment is largely done with dedicated assessment formats, which are aimed at capturing specific learning objectives. The results of assessments can be considered the observable *learning outcome* of competency-based education and are defined in terms of knowledge, skills, and behavior. Intended learning outcomes are preferably described with action verbs, which indicate the required cognitive level. Furthermore, the conditions under which the concrete behavior is expected to be demonstrated, must be specified in the intended learning outcomes [27,28]. Examples of intended learning outcomes for a content domain and for a generic skill are given below (Section 5). Learning outcomes can be ordered in different domains and different developmental stages to guide curriculum development.

In the previous paragraph the term ‘learning outcome’ is used in a specific sense to describe the results of assessment of the knowledge and skills elements of individual competencies; a learning outcome in this case is subordinate to a competency. It must be remarked that in the literature the term ‘learning outcome’ is also used in a more general way to describe the results of an educational program at different levels of integration; acquired competencies and entrusted professional activities can also be described as learning outcomes [15,22].

## 4. Curriculum Design Process

**Figure 1:**
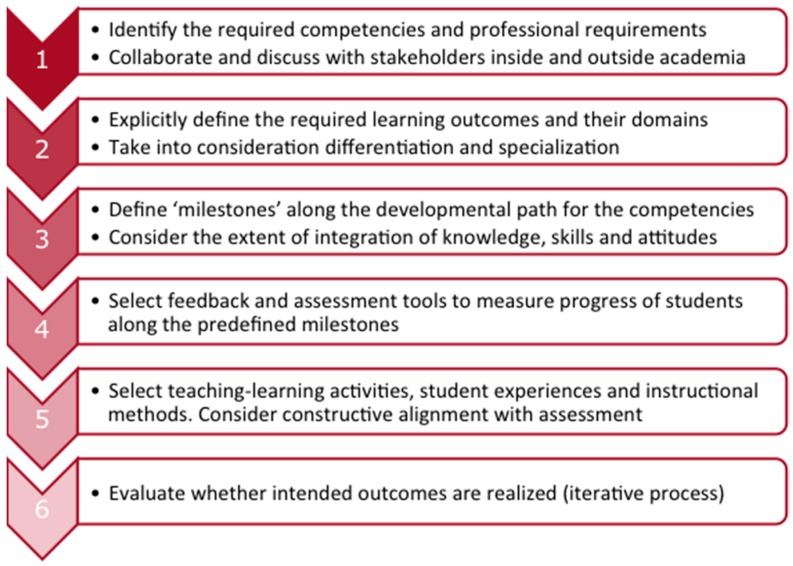
The curriculum design process.

The design of a competency-based curriculum ideally follows a specific sequence from competencies to learning outcomes, to assessments, to teaching-learning activities. This process can be described in six steps (Figure 1, adapted from [3]). Depending on the local situation, a curriculum change process can be more or less challenging, and success or failure will depend on the felt sense of urgency, the creation of a shared explicit vision on the future, and the willingness of all participants to engage in discussing fundamental issues, related to scientific identity and societal responsibility. Involvement of a diversity of stakeholders, both within and outside academia, and a careful ‘orchestration’ of the change process is necessary [29,30]. In our experience a combination of strong external pressure (e.g., a critical visitation or a critical attitude of professional organizations), internal dissatisfaction with the existing educational quality (often latent among teachers, students, and alumni), and courage of the institutional leadership to make a fundamental change, will make transformation from a ‘traditional’ curriculum to a competency-based curriculum possible. Even then, it is advised to monitor the change process carefully and to be aware of the socio-political aspects of the way the change process is organized [29]. 

Once a decision is made to embark on the journey to CBPE, the first two steps (Figure 1) are mainly strategic and intend to position the curriculum in the local context. The first step can be complex because the pharmacy profession has evolved from a nearly exclusively product-orientation to a more patient-orientation. Within a faculty or department a certain degree of consensus must be reached on the consequences of this shift, which necessitates more attention to softer disciplines such as pharmacotherapeutics and patient counselling, including communication skills. A pitfall in the first curriculum development step can be the introduction of new disciplines and new skills without reducing more traditional ones, resulting in overburdening the curriculum. Moreover, the main driving force for a curriculum rebuilding must be the learning process of the students and the responsibility to educate them to competent professionals, who can function adequately in the context of the local health care system or the local pharmaceutical research environment [16,31]. This means that—even though competency frameworks can be used as guidelines—interpretation and fine-tuning of the required competencies and competence levels is necessary. Another aspect is the need to consider accommodating a certain degree of specialization or profiling within the curriculum. The result of the strategic choices made will be a description of competencies and learning outcomes, which is more detailed than the general framework used as a starting point. Several examples of curriculum implementations in different contexts can be found in the literature (see Table 1).

- **Tip 1: Use a competency framework.** Several competency frameworks are available (see Appendix A). All can be used as a starting point for curriculum development but interpretation and fine-tuning to the local situation is necessary.
- **Tip 2: Consult all your stakeholders.** In designing a new curriculum consultation of the outside world is necessary to align the competences of recent graduates to the local professional and healthcare needs.
- **Tip 3: Think forward (scenarios).** Curriculum changes are usually implemented gradually, starting from the first year of the program. This means, that your newly-educated graduates will enter practice at least five years from now!

## 5. Curriculum Construction

Step 3 of the curriculum implementation process (Figure 1) is a crucial one. A competency-based curriculum is much more than a collection of courses: curricular elements, such as individual courses, (research) projects and pharmacy practice placements need to be organized in a logical sequence and decisions must be made about the obligatory or elective nature of the elements, taking into account possible specializations or profiling of students during the curriculum. It is helpful to explicitly formulate principles for the curriculum construction, which can serve as an internal ‘frame of reference’ or ‘reflection tool’ for steering and adjusting the construction process. Sharing these principles with teachers, students, and others involved in the curriculum implementation can ease the development process. In Table 2 an example is given of the principles that we have used during the curriculum design process at Utrecht University in the past.

Two aspects of the curriculum design need further attention: integration of content and skills in curricular elements and the longitudinal development of knowledge and skills, also described as horizontal and vertical integration, respectively [11]. The first aspect—integration of knowledge and skills—is a fundamental requirement in CBPE because students are expected to acquire complex competences during their study, where the required knowledge, cognitive and non-cognitive skills are expected to be used in an integrated way (AACP 2013, AFPC 2010, [11]). For the design of a competency-based curriculum this raises the question where, when, and how integration can be realized. In traditional curricula the change from non-integrated to integrated learning can be very abrupt, usually when a student is confronted with pharmacy practice for the first time, either during rotations or entry into professional practice. In less traditional curricula a more gradual approach, where students are moving from learning skills in isolation to application of skills in the context of professionally relevant tasks—with a gradual increase in complexity—is advocated [11,39]. This can be achieved by using problem-based and project-based learning methods of a relatively restricted nature in early phases of the curriculum, and a gradual increase in the complexity of assignments or projects as the curriculum progresses [12]. In later stages of the curriculum, simulations of pharmacy practice (e.g., the pharmacy game Gimmics^®^, [40]) and organizing the curriculum around EPAs (see above) can train students in real-life pharmacy practice situations under complex, but still safe and supervised, conditions without giving students full responsibility.

A gradual increase in the extent of integration of skills as the curriculum progresses requires that the development of skills and their integration with the content of the curriculum is explicitly analyzed and translated into teaching and learning activities, which confront students with challenging tasks during the whole curriculum. This requires that knowledge about the learning of skills must be present among the teachers and that some overarching description is available of the way development of skills is organized, monitored and assessed. In our situation in Utrecht this is realized by making selected teachers responsible for the development of different skills tracks, such as ‘pharmaceutical calculations’, ‘compounding’, ‘research methodology’, ‘oral communication’, and ‘written communication’. These teachers are stimulated to specialize in these didactic areas and participate in local networks with teachers from other faculties or universities. Within the pharmacy program, they function as consultants to the teachers who are responsible for the different courses of the curriculum. Similar track or stream coordination functions have been described for other curricula [31,41,42]. 

**Figure 2:**
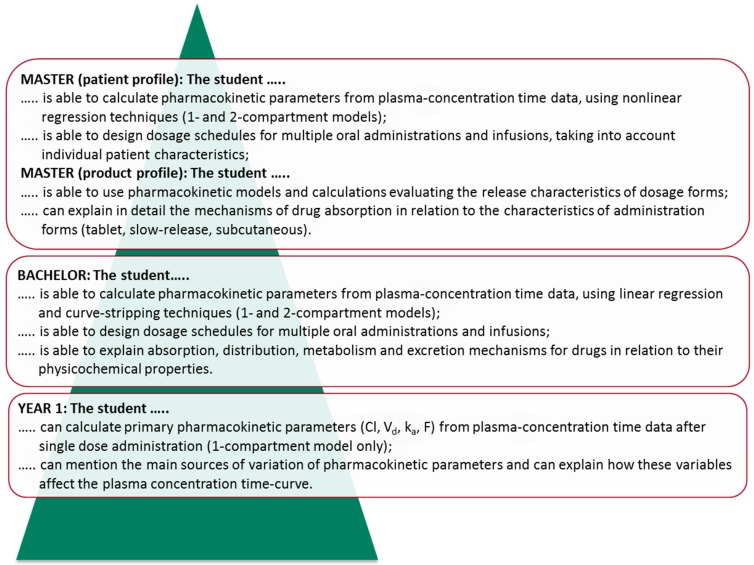
Example of curriculum layers for a content domain. Learning outcomes for the domain ‘pharmacokinetics’ at intermediate stages of the pharmacy curriculum in Utrecht. In this curriculum nine different content domains are distinguished, and learning outcomes are specified for the end of year one, for the bachelor degree, and for the master degree.

**Figure 3:**
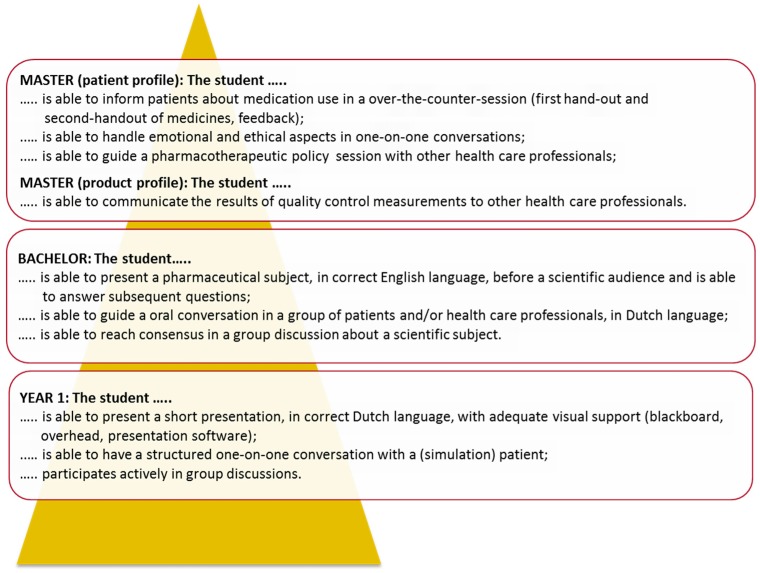
Example of curriculum layers for a skills domain. Learning outcomes for the skill ‘oral communication’ at intermediate stages of the pharmacy curriculum in Utrecht. In this curriculum nine different skills domains are distinguished in total, and learning outcomes are specified for the end of year one, for the bachelor degree, and for the master degree.

Analogous to the progression of skills, the development of content knowledge in the curriculum requires an explicit analysis of the way knowledge in different curricular domains is built up during the curriculum. These analyses can be used to explicitly formulate learning outcomes, which students are expected to have reached at intermediate stages of the curriculum. Once these intermediate stages (or ‘milestones’) are described, they can be used to inspire student assessment formats and guide the definition of actual course content on different levels of the curriculum. Examples of explicit intended learning outcomes at intermediate stages (end of year one, bachelor degree, and master degree) for a content domain and a skills domain of a curriculum are given in Figure 2 and Figure 3. In the example of the content domain ‘pharmacokinetics’ (Figure 2), the gradual built up of knowledge from basic concepts to practice-oriented applications is illustrated. In the example of the skills domain ‘oral communication’ (Figure 3), it can be seen that the requirements gradually increase in complexity and that some profiling is specified during the master phase.

Designing a curriculum is essentially a creative process, which requires the contribution of variously-minded individuals, and is best done with a combination of teachers, students, educational specialists, and administrative support personnel. Both creative, bird-like, leaders and meticulous, ant-like, workers are needed in different stages of the process [29]. In our experience, this can be organized as a curriculum committee with a flexible structure where sub-tasks can be allocated to smaller subsets of the committee as the need arises (see also [29]). Descriptions of available curriculum design processes may function as an inspiration for the reader [12,33,34,36].

- **Tip 4: Integrate content and skills as far as possible.** Skills can initially be trained in isolation, but must be integrated with course content as the curriculum advances. Professional activities usually require that knowledge, cognitive skills, and non-cognitive skills are used in an integrated way.
- **Tip 5: Appoint curriculum coordinators.** CBPE requires that the longitudinal development of knowledge and skills progresses gradually from relatively simple and isolated to more complex and integrated. This requires monitoring and readjustment of the curriculum structure by skills consultants and/or stream coordinators.

## 6. Student Assessment

In the next step of the implementation process (step 4 in Figure 1) formats for the summative and formative assessment of students are designed [43,44]. The goal of summative assessment (or: assessment *of* learning) is to evaluate and grade students at the end of the different curricular elements by comparing it to some standard or benchmark. The overall purpose of summative assessments in a curriculum is to guarantee that each individual student has fulfilled the curricular requirements. In the context of CBPE this means that the total of summative assessments is supposed to be representative for all required competencies. As a consequence, the student can be considered ‘competent’ at the level specified by the description of the required degree competencies. 

The goal of formative assessment is different. Formative assessment (or: assessment *for* learning) is intended to monitor student learning, and to inform teachers and students about progress in the learning process. Formative assessment essentially has a feedback purpose and can help students to identify their strengths and weaknesses and to identify areas that need additional attention. The results of formative assessments can help teachers to identify areas which appear to be problematic for students, and can help them to adapt and improve their teaching.

**Figure 4:**
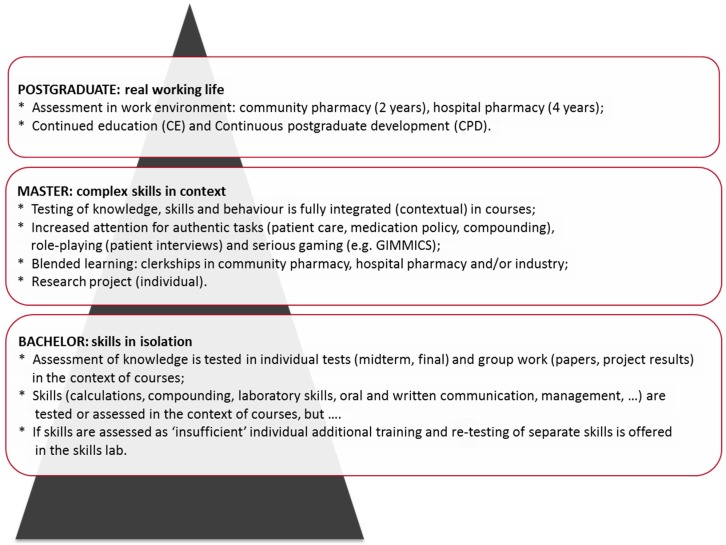
Example of curriculum layers for assessment of skills. Assessment formats in the curriculum ideally should move from simple, isolated assessments to more integrated, complex assessment formats. In this example the assessment principles of the pharmacy curriculum in Utrecht, including subsequent postgraduate education, are given as an example.

Designing assessment tasks, which have a clear relation to the required competencies in CBPE, is a challenging task [22,45]. As the focus in CBPE, compared to more traditional educational formats, is strongly emphasizing the development of student abilities [3], authentic assessment tasks are called for. Authentic assessment tasks mimic aspects of the future professional life of the students and can greatly contribute to student motivation. As the curriculum progresses, assessment tasks can increase in complexity to maintain consistency with the gradual evolution of the curriculum in the direction of professional identity (illustrated in Figure 4; see also [16,24,25]). In order to maintain student motivation and to prevent student burnout, overburdening the curriculum with multiple summative assessments should be prevented. In our experience it is better to concentrate on a limited number of well-chosen summative assessments, and invest more in frequent formative assessments. Spreading assessment periods over the study year and making assessment an integral part of curricular elements (courses, projects, rotations) results in a system of ‘continuous assessment’, which improves study behavior and minimizes test anxiety and student burnout. Investing in the development of formative assessment tasks emphasizes the function of assessment-for-learning (formative), rather than the function of assessment-of-learning (summative) [44,45].

Several assessment formats have been developed for formative assessment in competency-based education, such as serious games [40], and tools for self-evaluation and reflection, such as portfolios [46,47]. Objective structured clinical examinations (OSCEs, [48]) and an internet-based assessment tool for the assessment of advanced pharmacy practice experiences [18] can be used for summative assessment. It is beyond the scope of this article to fully evaluate the range of available assessment tools and their use in CBPE (but see [22] for a recent overview of the issues involved).

- **Tip 6: Less is more, in particular for summative assessment.** A pharmacy curriculum is easily overburdened; this can lead to burnout of students and teachers. Restrict contact hours and high-stakes examinations to a well-chosen minimum; concentrate on non-summative feedback.
- **Tip 7: Use authentic assessment tasks.** Authentic learning activities and assessment tasks (cases, OSCE), simulations (serious gaming) and the use of entrustable professional activities (EPAs) can motivate students and can prepare them for their professional life.

## 7. Effective Learning and Constructive Alignment

Competency-based education heavily relies on constructivist psychological principles, in which educational methods focus on the learning of students [27,49,50], where students construct meaning from what they do during their learning activities. In step 5 of the curriculum design process (Figure 1), the role of the teachers is to design the teaching-learning environment (TLE) in such a way that the student cannot escape from learning. In order to reach this goal all aspects of the TLE needs to be carefully designed. The learning of students is not only influenced by their perception of the assessment tasks (see above), but also by the way teaching is delivered, by the teacher behavior, and by the rules and regulations which pertain to the curriculum. The principles of *constructive alignment* [27,31,51] can be used to align all aspects of the TLE as good as possible. Several examples of carefully designed pharmacy curricula are described in the literature (see Table 1).

It is recommended to use an explicit, evidence-based, educational model to guide the development of learning tasks and design principles for a curriculum (see Table 2 for an example). Once formulated, the model can be used to make argued choices for teaching and learning activities, assessment of students and organizational aspects, whenever discussions arise during the actual implementation of the curriculum. Having an explicit model for the learning process will also protect against taking potentially counterproductive measures (see [27], pp. 309–315).

**Figure 5:**
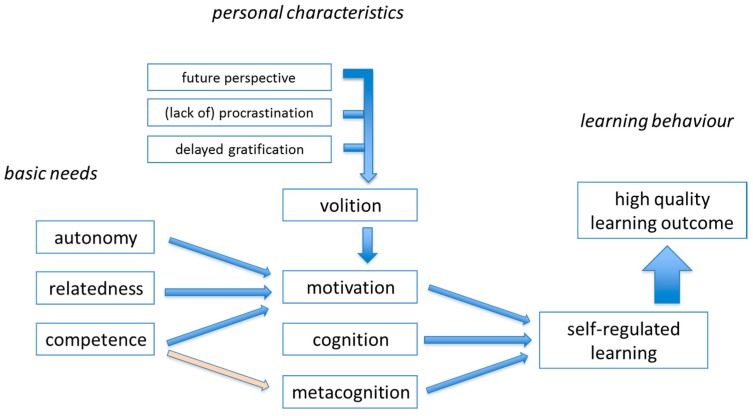
A model for effective learning.

Effective TLEs with high-quality learning outcomes need to be designed in such a way that students are motivated for deep, self-regulated, learning [31,44,52]. Several aspects of a model for effective learning are summarized in Figure 5. Extensive educational research has shown that—in addition to cognitive capacity—personality characteristics, motivational aspects, and teacher behaviors can contribute to the quality of learning [27,52]. Autonomous motivation, in contrast to controlled motivation, can contribute to high-quality outcomes [52,53]. As explained by the self-determination theory, student motivation is enhanced by giving students *autonomy* in studying and by creating opportunities to develop *relatedness* to fellow students and teachers, in addition to paying attention to the development of *competence* [52]. Problem- and/or project-based learning are educational methods, which are well-aligned with the development of the autonomy, relatedness and competence elements of this educational model [27,31]. Designing challenging student tasks and explicit attention for *reflection on learning* also will enhance the quality of learning outcomes. Case-based learning, for example, can be very effective for studying pharmacotherapy-oriented tasks and for practicing patient- and physician-directed communication. It is beyond the scope of this paper to describe all aspects of designing effective TLEs; excellent literature sources are available [12,27,51,52]. 

A newly-developed curriculum is seldom ideal from the start [54], and several years may be necessary to improve upon the original design. As a final step of the curriculum design process (Step 6 in Figure 1) a cycle of curriculum evaluation and refinement is needed. Both short-term and long-term feedback loops are necessary. In the short-term feedback loop all curricular elements (e.g., courses) are evaluated on a regular, usually annual, basis. In the long-term feedback loop the curriculum is undergoing review every five to ten years. This review may be synchronized with external evaluations or visitations, but is preferably also done internally.

Evaluation of the curriculum *as a whole* is usually done by mapping the curriculum using an existing framework [42,55]. Curriculum mapping can serve different purposes, but in the context of this paper the main purpose will be providing guidance in further improvement of the curriculum, as described by Farris et al. [30] and Zelenitsky et al. [56]. Mapping the *experienced curriculum* (the curriculum as perceived by the students) on the *intended curriculum* (the designed curriculum) can be very useful for identifying gaps, overlaps, and discontinuities in the curriculum construction [55,57].

- **Tip 8: Adopt frameworks for cognitive and skills development.** An explicit, evidence-based, educational model can guide choices for assessments, learning tasks, and can protect against counterproductive measures.
- **Tip 9: Use curriculum mapping for internal quality enhancement.** Mapping the various curricular elements (course, etc.) against existing frameworks can be very helpful in identifying curricular gaps, overlaps, and discontinuities.

## 8. Management and Quality Enhancement

When a new curriculum is designed, a continuous process of refinement and optimization is started. This is a long-term and laborious process, which may last several years [30,54] and requires an effective quality management system [58]. It is advised that continuity for this process is organized at the highest possible organizational level (faculty, institute) and that the adopted design principles (Section 5) and an explicit educational model (Section 7) are used as an internal ‘frame of reference’ to guide all discussions with the involved teachers, students, and other stakeholders. Open-minded and frequent communication with everybody involved is necessary to prevent misalignment of curricular elements and to assure that the delivered curriculum (the curriculum as presented to the students) is as close as possible to the designed curriculum. It is advised to use evaluation- and feedback-cycles at both the course level and the curriculum level (see above) to maintain flexibility and adaptability [58].

Integration of curricular disciplinary content, integration of knowledge and skills, and the use of novel assessment formats require that some teachers are given the opportunity to pay attention to these aspects of the curriculum, preferably on a curriculum level (i.e., under direct responsibility of a director of education or a curriculum manager). In this way the consistency of educational approaches in different curricular elements (across courses) can be improved. Appointment of stream coordinators, skills consultants, or specialization of teachers in novel assessment methodology is called for [59]. Another potential new teacher role is the role of a tutor, who can advise students in their personal development. All these non-traditional roles are preferably organized as temporary part-time tasks besides a primary role as teacher, responsible for delivering disciplinary content in the curriculum. By organizing non-traditional roles in this way, the connection with other teachers and flexibility of the organization can be maintained as good as possible. We strongly advise against a strict separation between teachers having traditional and non-traditional roles in the organization of the curriculum. 

New roles for teachers require development of new educational expertise and the introduction of a trajectory for continuous professional development *as a teacher* [60]. This can be organized in a more or less structured way, ranging from formal training in teaching methodology [59], to a personal development trajectory for future program leaders [61,62]. Suggestions for the collaborative development of specific expertise can be found in the educational literature [63]. Depending on the local situation (such as size of a faculty or institution, number of teachers involved, existing university policy) educational development programs can range from informal, small-scale initiatives to relatively large-scale formal training and development programs [64]. Engaging in a scholarly approach to teaching and learning (SoTL), involving reflection on teaching experiences, use of educational research literature, and evidence-based development of teaching, can contribute to the quality enhancement of CBPE [65,66]. In our experience, the content and scale of training or development activities should be carefully adapted or ‘titrated’ to the needs felt by teachers [59,65]. Effective development programs usually involve a combination of individual and collaborative projects, sharing of knowledge and experiences, interaction with other like-minded teachers, and goal-directed development of educational innovations [61,64].

- **Tip 10: Assure management continuity.** Development and optimization of CBPE requires a long-term perspective and continuity in the educational development. This is best achieved by appointing a director of education and/or by forming a curriculum management team.
- **Tip 11: Develop educational expertise and specialization.** A competency-based curriculum requires teachers to develop expertise in the fields of autonomy-supportive teaching and competency assessment.
- **Tip 12: Develop scholarship of teaching and learning (SoTL).** Building a competence-based curriculum requires the development and testing of non-standard teaching-learning activities and novel assessment formats. Teachers and curriculum developers can benefit from a scholarly approach, using educational literature, exchange of good practices and training or coaching in (inter)national networks.

## 9. Summary and Conclusions

Implementing CBPE is a time-consuming and complicated process, which requires ‘translation’ of formulated competencies into intended learning outcomes and assessment formats. Conscious choices and decisions on all organizational levels are needed to achieve consistency between learning tasks, feedback to students, teacher roles, and organization of the curriculum. Formulating design principles and adopting an explicit educational model, based on evidence-based educational psychology, can be helpful in guiding curriculum development and optimization. Finally, the institutional management structure should support the required human resources allocation, which involves training of teachers for new roles and the stimulation of teacher professional development.

## Figures and Tables

**Table 1:** Examples of curriculum design and construction.

Curriculum	Description	Reference
B.Sc.	Content and generic skills for a pre-professional curriculum (nationwide, USA)	[32]
B.Pharm.	Design of an outcomes-based Pharmacy curriculum (Hong-Kong, China)	[33]
B.Pharm.Sc.	Undergraduate honours programme for the training of pharmaceutical researchers (Utrecht, the Netherlands)	[12]
Pharm.D.	An integrated professional pharmacy curriculum (Denver, USA)	[34]
B.Sc. + M.Sc.	Design of a complete bachelor and master programme (Helsinki, Finland)	[31]
Ph.D.	Research training for clinical pharmaceutical sciences: assessments and rubrics (Pittsburgh, USA)	[35]
M.D.	Content and skills for the core curriculum of a medical school (Sheffield, United Kingdom)	[36]
M.D.	Teaching, training, and assessment of professional behaviour in medicine (Amsterdam, The Netherlands )	[37]
Physician assistants	Teaching, training and assessment for physician assistants (Utrecht, The Netherlands)	[38]

**Table 2:** Seven principles for design and construction of a curriculum.

1	The curriculum is designed as a coherent program.
2	The program stimulates active study behaviour, is challenging and varied.
3	Acquisition, application and integration of knowledge and skills take place in a context relevant for the future profession.
4	Within the program systematic and explicit attention is paid to the development of academic and personal skills and values.
5	Direction of the learning process is gradually shifted from teacher to student.
6	The program enables students to follow individual interests by offering elective courses and a patient- or product-oriented profile.
7	A well-balanced system of mentoring and assessment is used, which takes into account the steering effects of testing.

Example of guiding principles used for the design of a new pharmacy curriculum (bachelor, master) at Utrecht University in 2001, cited from [13].

## Appendix A. Competency- or Outcome-Based Frameworks

AACP 2013. CAPE Educational outcomes, American Association of Faculties of Pharmacy (AACP), Chicago 2013. Available online at www.aacp.org/resources /education/cape/Pages/default.aspx (last accessed on 8 February 2017).

AFPC 2010. Educational Outcomes for First Professional Degree Programs in Pharmacy (Entry to Practice Pharmacy Programs) in Canada. Association of Faculties of Pharmacy of Canada (AFPC), Vancouver 2010. Available online at http://www.afpc.info/node/39 (last accessed on 8 February 2017).

EPCF 2016. European Pharmacy Competency Framework, The Phar-QA Quality in Pharmacy Education in Europe consortium and European Association of Faculties of Pharmacy (EAFP), Brussels and Malta 2016. Available online at eec-pet.eu/pharmacy-education /competency-framework/ (last accessed on 8 February 2017).

FIP 2012. A global framework for services provided by Pharmacy workforce. International Pharmaceutical Federation (FIP), The Hague, 2012. Available online at www.fip.org /files/fip/ PharmacyEducation/GbCF_v1.pdf (last accessed on 1 December 2016).

GPhC 2011. Standards for the initial education and training of pharmacists, General Pharmaceutical Council (GPhC), 2011. Available online at www.pharmacyregulation.org/sites/default/files /GPhC_Future_Pharmacists.pdf (last accessed on 1 December 2016).

NCSF 2010. National Competency Standards Framework for Pharmacists in Australia, Pharmaceutical Society for Australia (PSA), 2010. Available online at www.psa.org.au/ downloads/standards/competency-standards-complete.pdf (last accessed on 1 December 2016).
